# ICTV Virus Taxonomy Profile: Poxviridae 2023

**DOI:** 10.1099/jgv.0.001849

**Published:** 2023-05-17

**Authors:** Colin J. McInnes, Inger K. Damon, Geoffrey L. Smith, Grant McFadden, Stuart N. Isaacs, Rachel L. Roper, David H. Evans, Clarissa R. Damaso, Olivia Carulei, Lyn M. Wise, Elliot J. Lefkowitz

**Affiliations:** 1 Moredun Research Institute, Penicuik, UK; 2 Centers for Disease Control and Prevention, Atlanta, USA; 3 University of Oxford, Oxford, UK; 4 Arizona State University, Tempe, USA; 5 Perelman School of Medicine at the University of Pennsylvania, Philadelphia, USA; 6 East Carolina University, Greenville, USA; 7 The University of Alberta, Edmonton, Canada; 8 Cidade Universitária da Universidade Federal do Rio de Janeiro, Rio de Janeiro, Brazil; 9 University of Cape Town, Cape Town, South Africa; 10 University of Otago, Dunedin, New Zealand; 11 UAB School of Medicine, Birmingham, USA

**Keywords:** ICTV Report, taxonomy, *Poxviridae*, *Chordopoxvirinae*, *Entomopoxvirinae*

## Abstract

*Poxviridae* is a family of enveloped, brick-shaped or ovoid viruses. The genome is a linear molecule of dsDNA (128–375 kbp) with covalently closed ends. The family includes the sub-families *Entomopoxvirinae*, whose members have been found in four orders of insects, and *Chordopoxvirinae,* whose members are found in mammals, birds, reptiles and fish. Poxviruses are important pathogens in various animals, including humans, and typically result in the formation of lesions, skin nodules, or disseminated rash. Infections can be fatal. This is a summary of the International Committee on Taxonomy of Viruses (ICTV) Report on the family *Poxviridae*, which is available at ictv.global/report/poxviridae.

## Virion

**Fig. 1.**
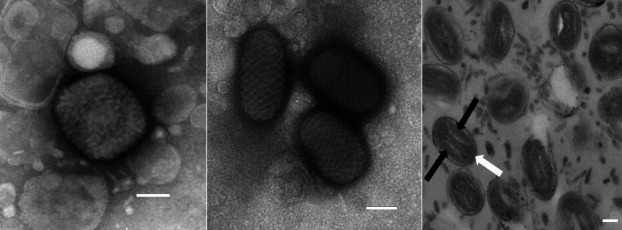
Virion morphology. Electron micrographs of negatively-stained preparations of an orthopoxvirus mature virion (left); parapoxvirus mature virions (centre); and avipoxvirus virions displaying two lateral bodies (black arrows) either side of the ‘dumbbell’ core (white arrow) (right). Bars, 100 nm. Images courtesy of David J. Everest, Animal and Plant Health Agency (APHA), Surrey, UK.

 Virions are brick-shaped (220–450 long × 140–260 wide × 140–260 nm thick) with a lipoprotein surface membrane displaying tubular or globular units (10–40 nm). Virions can also be ovoid (250–300 long × 160–190 nm diameter) with a surface membrane possessing a regular spiral filament (10–20 nm in diameter) (Table 1, Fig. 1). Negatively-stained electron microscopy images show that the surface membrane encloses a biconcave or cylindrical core that contains the DNA genome and proteins organized in a nucleoprotein complex. One or two lateral bodies appear to be present in the concave region between the core wall and membrane [1].

**Table 1.** Characteristics of members of the family *Poxviridae*

Example:	vaccinia virus (AY243312), species *Vaccinia virus*, genus *Orthopoxvirus*
Virion	Generally oval or brick-shaped, 220–450×140–260×140–260 nm with a lipoprotein surface membrane displaying tubular or globular units or a regular spiral filament
Genome	Single linear molecule of dsDNA with covalently closed ends; 128–375 kbp
Replication	Cytoplasmic. Mediated by virus-encoded proteins with self priming. DNA is replicated as long concatemers resolved by a viral HJ endonuclease
Translation	From polyadenylated, capped mRNA transcripts, synthesized from both DNA strands by virus-encoded RNA polymerase
Host range	Vertebrates and arthropods
Taxonomy	Realm *Varidnaviria*, kingdom *Bamfordvirae*, phylum *Nucleocytoviricota,* class *Pokkesviricetes,* order *Chitovirales*: 2 subfamilies, >20 genera and >80 species

## Genome

**Fig. 2.**
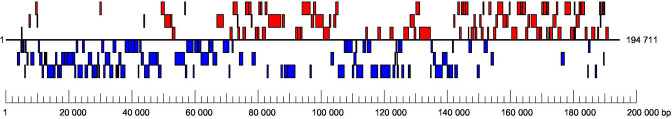
Schematic representation of the vaccinia virus Western reserve genome (AY243312) . Genes generally do not overlap, even when on different strands, giving a coding density of around 90 %.

The poxvirus genome comprises a linear molecule of dsDNA (128–375 kbp, Fig. 2), with covalently-closed termini; terminal hairpins constitute two isomeric, imperfectly paired, ‘flip-flop’ DNA forms consisting of inverted complementary sequences. The genomes generally encode more than 128 genes. A set of about 49 genes, conserved across all species, encode proteins essential to the transcription and replication of the genome. Species-specific genes encode proteins that confer a replicative advantage in their hosts, for example by subverting the host immune response to infection [2,3] or providing cell-specific growth factors.

## Replication

Poxvirus entry is at the plasma membrane or following endocytosis and involves a complex of many virus-encoded proteins [4]. The early phase of replication includes expression of proteins needed for replication of viral DNA, followed by expression of structural protein genes [5]. The assembly of virus particles is multistage and results in the exocytosis of enveloped virions. DNA replication and gene expression occur in the cytoplasm in ‘viroplasms’ or ‘virus factories’.

## Taxonomy

Current taxonomy: ictv.global/taxonomy. The family *Poxviridae* includes two subfamilies: *Chordopoxvirinae* and *Entomopoxvirinae*. Members of *Chordopoxvirinae* infect vertebrates (mainly mammals, birds, reptiles and fish) and are classified into the genera *Avipoxvirus*, *Capripoxvirus*, *Centapoxvirus*, *Cervidpoxvirus*, *Crocodylidpoxvirus*, *Leporipoxvirus*, *Macropopoxvirus*, *Molluscipoxvirus*, *Mustelpoxvirus*, *Orthopoxvirus, Oryzopoxvirus*, *Parapoxvirus*, *Pteropopoxvirus*, *Salmonpoxvirus*, *Sciuripoxvirus*, *Suipoxvirus, Vespertilionpoxvirus* and *Yatapoxvirus*. Members of *Entomopoxvirinae* infect invertebrates and are classified into the genera *Alphaentemopoxvirus*, *Betaentemopoxvirus*, *Deltaentomopoxvirus* and *Gammaentemopoxvirus*; members of each genus infect a different order of insect.

Poxviruses include human pathogens such as variola virus (the aetiological agent of smallpox, an eradicated disease), and economically important animal pathogens such as fowlpox virus and lumpy skin disease virus [6].

## Resources

Full ICTV Report on the family *Poxviridae*: ictv.global/report/poxviridae.
